# Cochlear implant radiography: technique adapted into a portable apparatus

**DOI:** 10.1590/S1808-86942012000100005

**Published:** 2015-10-20

**Authors:** Flávio Donizeti Molezini, Silvio Garcia Meira Junior, Domingos Lamônica Neto, Orozimbo Alves Costa Filho

**Affiliations:** a Technical Program in Radiology (Radiology Technician – Craniofacial Anomalies Rehabilitation Hospital – University of São Paulo); b MD. Radiologist (Collaborator of the Audiologic Research Center - (HRAC/USP)); c MD. Otorhinolaryngologist (Surgeon of the HRAC/USP, Bauru/SP); d Senior Associate Professor (Full Professor – University of São Paulo). Craniofacial Anomalies Rehabilitation Hospital – University of São Paulo (HRAC/USP)

**Keywords:** cochlear implantation, diagnostic equipment, radiography

## Abstract

The unavailability of advanced imaging equipment in the operating rooms of most hospitals - as well as the fundamental importance for surgeons of immediate observation of the cochlear implants soon after their insertion - makes conventional radiography a good option.

**Objective:**

To describe a practical, fast and low-cost radiographic method, allowing for evaluation of the electrodes regarding their position and integrity, during the insertion of cochlear implants.

**Materials and Methods:**

Radiographies from 262 cochlear implant patients were analyzed, from March 2005 to October 2008, by means of intraoperative radiography, soon after electrode insertion. All radiographies were analyzed by the surgeon in the intraoperative period and, afterwards, by the radiologist.

**Results:**

A total of 524 radiographies were analyzed, and 95.61% presented adequate technique - with the patient being positioned into the technique proposed in this study - as well as a clear visualization of the electrodes, regarded as satisfactory. On the other hand, 4.39% presented inadequate technique and/or unsatisfactory visualization of the electrodes, regarded as unsatisfactory.

**Conclusion:**

Although the portable X-ray apparatus presents limitations, the employment of proper techniques and accessories makes possible the obtainment of satisfactory radiographies to observe cochlear implants.

## INTRODUCTION

Considering the unavailability of the surgical arch digital fluoroscopy device – which is an advanced image acquisition system, offering real time imaging of a number of anatomical structures in any possible angle, side or direction, broadly utilized to guide different types of surgery – in most hospitals, especially because of its high cost, one good option to visualize the cochlear implant immediately after its insertion is to use a portable X-ray machine, since it is much more accessible and almost any hospital has one. Although conventional radiography is very much used to visualize the cochlear implant, its use in the surgical table with the portable equipment provides some degree of complexity and requires special attention, especially concerning some details, such as: position adaptation, respecting the conditions and limitations, the employment of proper doses matching the equipment power and the use of proper accessories which may guarantee image quality.

One general rule says that when you radiograph any object which thickness is greater than 10 cm, it is mandatory to use the anti-scatter grate which, in this case, is fit to the radiograph frame, and which aim is to filter the scatter radiation, optimizing image quality. Nonetheless, the use of this accessory considerably limits the possibility of tilting the central beam, thus being advisable to perpendicularly guided it to the grate/film, and direct it to the center. In this study, this deficiency was corrected adapting the head position in the proper way, with the goal of avoiding the overlapping of images among the inner ear structures and the denser structures of the temporal bone, keeping the central beam perpendicular to the film/grate.

## MATERIALS AND METHODS

We analyzed 524 radiographs, from 262 patients submitted to cochlear implant in the period between March, 2005 and October of 2008. The two intraoperative radiographs were done immediately after electrode insertion. The present paper was approved by the Ethics in Research with Human Beings Committee, where the study was carried out, protocol # 181/2009-SVAPEPE-CEP.

All the analyzed radiographic exams were carried out with a UNIMAX portable x-ray machine, from SIEMENS, maximum power of 90 kV and 30 mA.

All the radiographs were analyzed by the surgeon during the procedure and by a radiologist, being classified in satisfactory or non-satisfactory, according to the following criteria:

### Image contrast and density

Radiographic density is defined as the *intensity of darkness in the image of a processed radiographic film.*

Radiographic contrast is defined as the *density difference in adjacent areas of a radiographic image.*

The factors which directly influence image density and contrast are “Kilovoltage (kV), Milliamperage (mA) and time of exposure”, adjusted in the X-Ray machine console. These factors must be set making sure that the images obtained are of the best possible quality and the patients be exposed to the least possible radiation dose.

### Image resolution or definition (sharpness)

The definition or resolution of a radiographic image *is shown by the clarity or sharpness of the more delicate structural lines and the borders of structures or tissues.* The lack of visible resolution is called “blurring” or “lack of sharpness”.

The main factors which influence the resolution/ sharpness of the radiographic image are: The size of the focal area, the focal distance – image receptor (DRFI), the object distance – image receptor (DORI) and patient movement.

The use of a small focus area, DFRI increase and DORI reduction, result in resolution increase; patient movement may be controlled by asking for the patient's cooperation and/or using radiotransparent guards.

### Image distortion

Distortion is a *deformation in the size or shape of the object* projected on the radiographic recording medium.

The primary factors which affect distortion are: DFRI, DORI, object alignment – image receptor and alignment/centralization of the central beam.

Using a correct DFRI, minimizing the DORI, making sure the object and the image receptor are properly aligned and properly aligning/centralizing the central beam in relation to the structure to be radiographed, it is possible to control the distortion of a radiographic image.

We used anti-scatter grate in all radiographic images, since conventional radiography has a rule which states that for objects which thickness is greater than 10 cm, one must use the anti-scatter grate^1^, ^2^, ^3^, this accessory is needed in skull x-rays.

### Radiographic technique

In order to see the cochlear implant at the time of its insertion, we carried out two radiographic views: The first is anteroposterior skull - AP (Transorbital) and the 2^nd^ is 45° side-oblique, with the central beam (CB) perpendicular to the film. Both techniques hereby described were adapted in order to do the x-rays in the surgical table, with the patient in dorsal decubitus.

Tables 1 and 2 depict the technical data regarding the image acquisition of the present study.

**Table 1:** Technical factors of the cranium AP view (Transorbital).

Film size: 18 × 24 cm, longitudinal direction
Focus-Film distance: minimum of 100 cm (see specification of the grate used)
Power: Range of 70-80 Kvp (adult), 65-70 Kvp (child)

* Use fixed anti-scatter grate.

**Table 2:** Technical factors associated with the 45° side oblique view.

Film size: 18x24 cm, in the longitudinal direction
Focus-Film distance: minimum of 100cm (see specification of the grate used)
Power: Range of 70-75 Kvp (adult), 60-70 Kvp (child)

* Use fixed anti-scatter grate.

### Cranial AP (Transorbital)

#### Patient position

**Figure 1:**
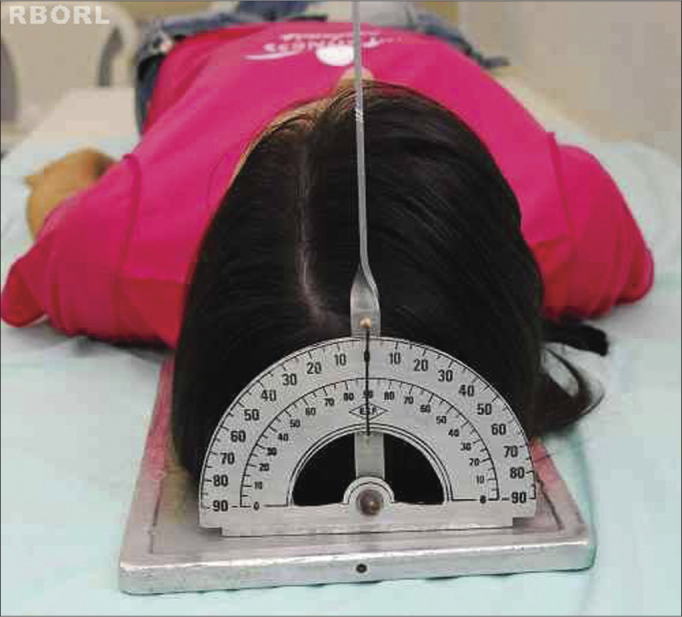
Positioning - median sagittal plane in 90° with the horizontal: transorbital (posterior view).

With the patient in dorsal decubitus, the frame is placed under the head, aligning the median sagittal plane in 90° with the horizontal plane, making sure that there is no head rotation and/or tilt (Figure 1).

**Figure 2:**
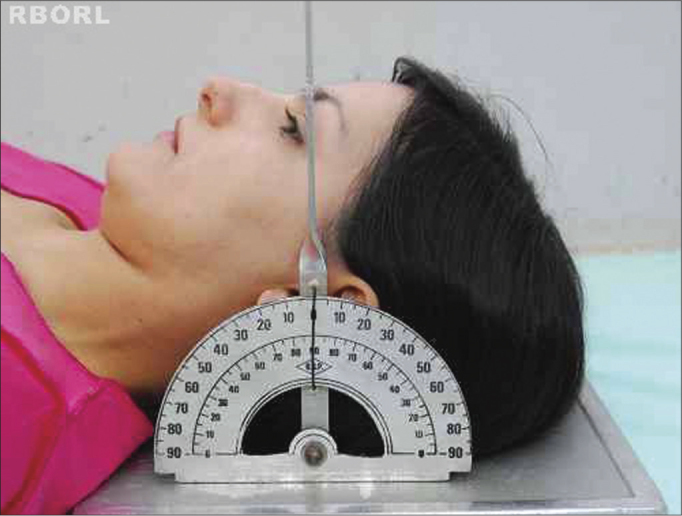
Positioning - meatal-orbital line perpendicular to the film: Transorbital (side view).

^*^ If needed, use radiotransparent guards in order to make sure the patient does not move during shooting.

Lightly flex the head, depressing the chin until the orbitomeatal line (OML) is perpendicular to the film (Figure 2).

#### Central Beam

Direct the CB perpendicular to the film/grate, parallel to the OML, directing it to the center of the orbit in the side of interest.

Centralize the film in regards to the projected Central Beam.

Collimate the skull external images or, if possible, use the extension cylinder. Should the patient be under mechanical ventilation, do no move him/her without the supervision of the anesthesiologist/surgeon in charge.

#### Expected radiological result

Proper positioning will project the bony labyrinth on the center of the orbit. The lack of skull rotation is shown by the same distance between the orbit margin and the lateral border of the skull in both sides and by the same distance of the median sagittal plane (identified by the crista Galli) to the external orbital margin in both sides^4^, ^5^.

**Figure 3:**
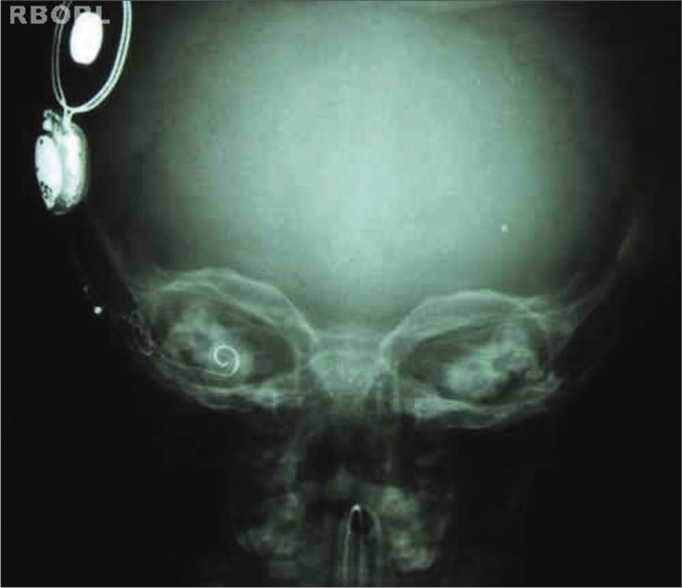
Expected radiological result (Transorbital AP).

**Figure 4:**
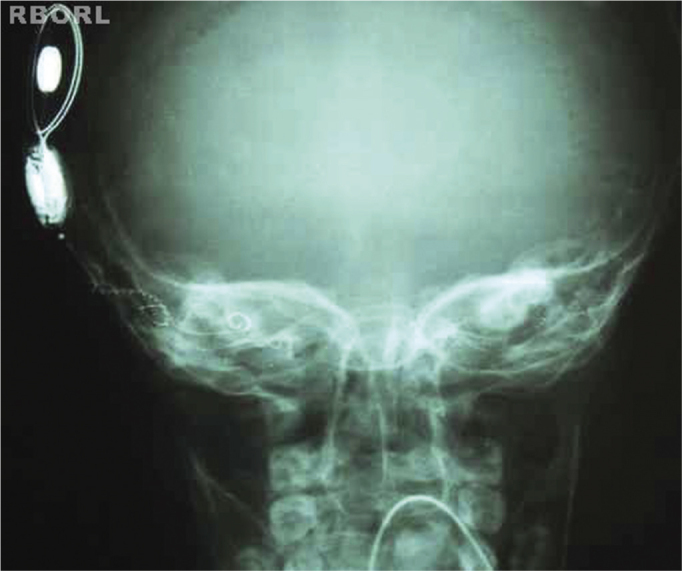
Expected radiological result (Transorbital AP).

Proper density and contrast will enable implant visualization in its entire extension, as well as the structures inside the bony labyrinth. Sharp bone margins show the lack of movement (Figures 3 and 4).

### 45° side-oblique view

#### Patient position

**Figure 5:**
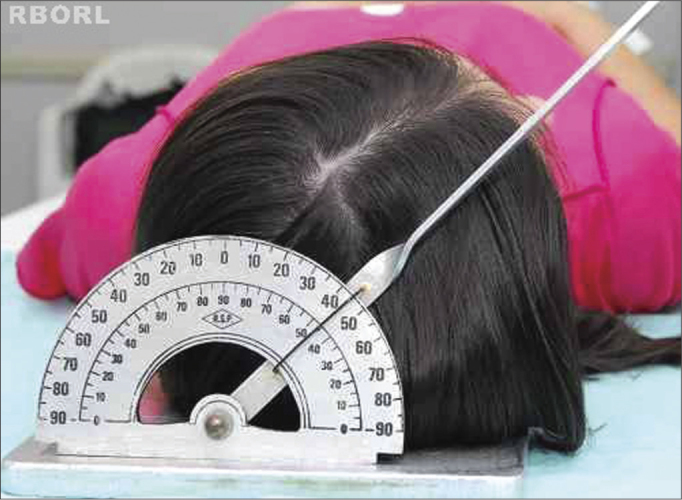
Positioning - 45° median sagittal plane with the horizontal plane: side oblique (posterior view).

With the patient in dorsal decubitus, the frame is placed under the head, turning the head sideways, towards the opposite side of interest until the median sagittal plane makes a 45° angle with the horizontal plane (Figure 5).

**Figure 6:**
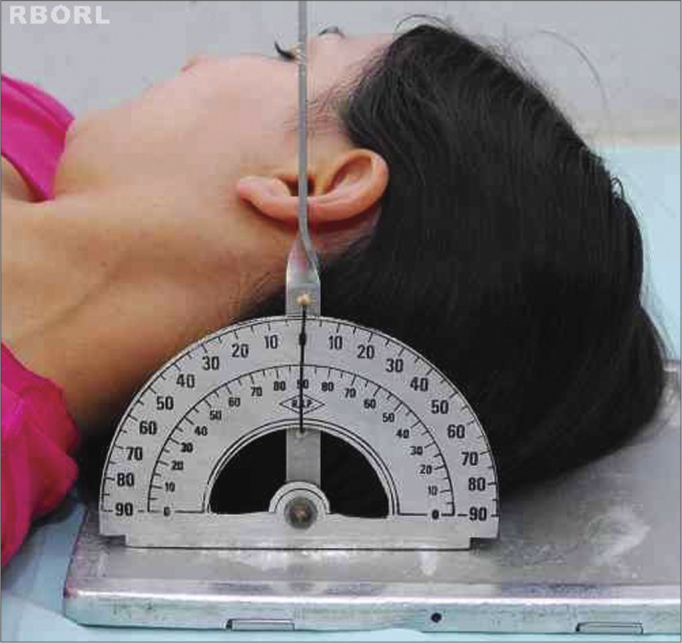
Positioning - meatal-orbital line perpendicular to the film: side oblique (side view).

To slightly flex the head, depress the chin until the OML is perpendicular to the film (Figure 6).

* If necessary, use radiotransparent guards to make sure there is not patient moving during the session.

#### Central Beam

Guide the CB perpendicular to the film/grate, the entry point will be 2 cm anterior to the external acoustic meatus at the elevated side.

Centralize the film to the projected Central Beam.

Collimate the petrous pyramid external margins at the raised side or, if possible use an extension cylinder.

OBSERVATION: If the patient is under mechanical ventilation, you should not move him/her without the supervision of the anesthesiologist and/or the surgeon responsible for the case.

#### Expected radiological result

A properly positioned image shall show the following:

Mandibular condyle superimposed on the neck spine. Bony labyrinth below the petrous crest. Posterior margin of the mandibular ramus overlapping the posterior margin of the neck spine^4^, ^6^.

**Figure 7:**
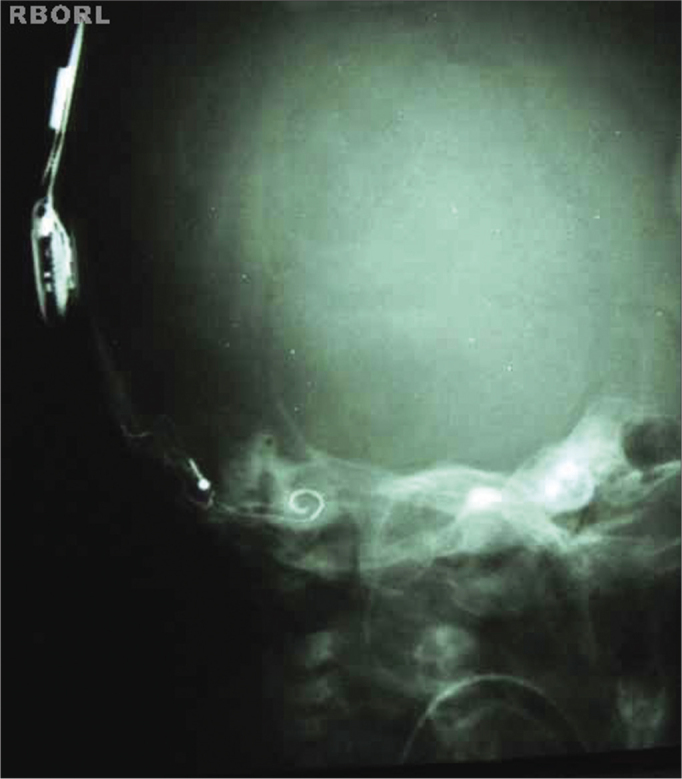
Expected radiological result (45° side oblique).

**Figure 8:**
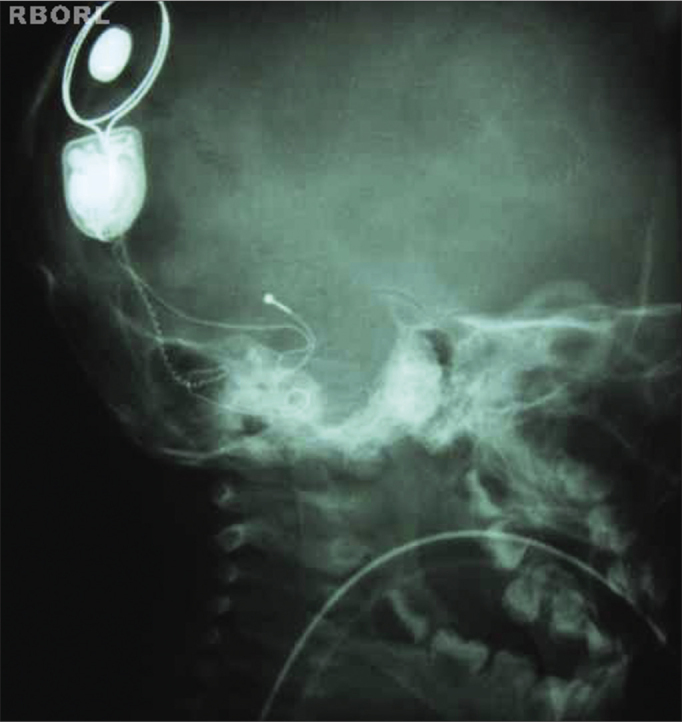
Expected radiological result (45° side oblique).

Proper density and contrast will enable the visualization of the entire implant, as well as all the structures inside the bony labyrinth. Clear bone margins show the patient was still (Figures 7 and 8).

## RESULTS

The surgeon, and later, the radiologist, assessed a total of 524 radiographies, which are listed on Table 3.

**Table 3:** Total number of radiographies assessed, separated by gender and side.

	Right ear	Left ear	Total
Males	94	172	266
Females	106	152	258
Total	200	324	524

Patient age range varied between 10 months and 46 years of age.

Of the total number of radiographies assessed, 501 (95.61%) were done under proper technique, with correct patient positioning, following the technique described in the present study, proper density and contrast, proper sharpness, enabling proper visualization of the cochlear implant electrodes and the temporal bone structures, being considered satisfactory.

Of the 524 radiographies assessed, 23 (4.39%) had some failure in terms of technique or positioning, inadequate density or contrast because of technical factors, such as dose selection or processing failure, or, still, inadequate visualization of the electrodes (because of distortion or lack of sharpness) being considered inadequate.

In the period studied, there were isolated cases (approximately 2%) in which there was a need for immediate surgeon intervention in order to reposition the electrodes, because of factors such as: False trajectories (e.g. introduction in the superior canal); electrode compression; partial introduction with extra-cochlear electrodes. Such factors could later stimulate the facial nerve, as well as impair and/or make it impossible to activate the cochlear implant.

## DISCUSSION

Upon radiographying the ear using the Stenvers method (45° side oblique), it is necessary to have a 12°tilt in the central beam in the cranial direction, and the patient's head must be positioned in such a way that the meatal infraorbital line (MIOL) is perpendicular to the film, with the patient in ventral decubitus. In the Arcelin or inverted Stenvers method, which is carried out with the patient in dorsal decubitus, the central beam must be tilted in 10° in the caudal direction and the patient's head must also be positioned with the MIOL perpendicular to the film. This will optimize visualization of the temporal bone structures^1^, ^2^, ^7^.

The hereby described technique was adapted to be used with a portable equipment, which requires some peculiar accessories, with the frame equipped with anti-scatter grate, which limits the possibility of tilting the central beam. In order to make up for this shortcoming and obtain a good result, we changed the patient's head position, in such a way that the meatal-orbit line (MOL) was perpendicular to the film/grate.

This study showed that, even using portable equipment, it is possible to obtain satisfactory quality radiographs to visualize the entire cochlear implant, as well as its position and electrode integrity, serving as an extremely important tool for the surgeon (since the electrode impedance telemetry tests, by themselves, do not rule out the possibility of the implant having a false trajectory), even enabling an immediate intervention which reduces costs and the risks associated with a later surgery/anesthesia, as we could see in some isolated cases (approximately 2%), in which such intervention was needed because of mal-positioning or compression of the cochlear implant electrodes.

### Alternatives

One efficient alternative would be to use a Mobile Fluoroscopic Digital System with the C arm (surgical arch), which is a device that emits x-ray-type ionizing radiation, capable of doing radiographies and fluoroscopies, made up by a C arch mounted on wheels, X-Ray generator, X-Ray tube, command unit, image intensifier and a TV system with mobile support, with digital image subtraction. The images obtained are shown in real time, in a monitor; therefore making this a very versatile device and broadly utilized in orthopedic surgery; vascular, neurological, gastrointestinal, urological and other types of procedures, especially to guide the placement of prosthesis, catheters and implants in general. The main advantage in relation to the use of a portable X-ray device is exactly the possibility for immediate visualization of the image, having no need to wait for processing. The main disadvantages are: its high cost; its relatively larger size, requiring a considerable space in the operating room; sometimes there is also the need to renovate and adapt the room and surgical tables in order to achieve the necessary views; and, still, the use of larger radiation doses, depending on the exposure time utilized.

## CONCLUSION

Intraoperative radiography of the cochlear implant is a fundamental tool because it enables the surgeon not only to assess the position, but also to evaluate electrode integrity, when allowing for immediate intervention, when needed. The use of a portable device proved to be fast, practical and low cost, affordable to most hospitals.
